# Characteristics of Pyomyositis at a Pediatric Hospital in Osaka, Japan

**DOI:** 10.7759/cureus.86325

**Published:** 2025-06-18

**Authors:** Chikahisa Higuchi, Dai Otsuki, Masato Kobayashi, Ayana Yamanaka, Daisuke Tamura, Seiji Okada, Hidehiko Kawabata

**Affiliations:** 1 Department of Orthopedic Surgery, Osaka Women's and Children's Hospital, Izumi, JPN; 2 Department of Orthopedic Surgery, Osaka Rehabilitation Hospital for Children, Osaka, JPN; 3 Department of Rehabilitation, Osaka Women's and Children's Hospital, Izumi, JPN; 4 Department of Orthopedic Surgery, Graduate School of Medicine, Osaka University, Suita, JPN

**Keywords:** hip joint, infection, japan, pediatric hospital, pyomyositis

## Abstract

Introduction

Pyomyositis, also known as tropical pyomyositis, has traditionally been considered more prevalent in tropical regions. The number of pediatric pyomyositis cases has been increasing in several temperate countries. In this study, we examined the characteristics of pyomyositis in a single pediatric hospital in Japan, a temperate country.

Methods

We analyzed 108 patients diagnosed with pyogenic arthritis, pyogenic osteomyelitis, and pyomyositis who were followed up at our hospital over a 32-year period. Data were collected on sex, age of onset, affected site, causative organism, and year of occurrence. The characteristics of pyomyositis were identified through comparison with pyogenic osteomyelitis and pyogenic arthritis.

Results

The number of patients with pyogenic osteomyelitis, pyogenic arthritis, and pyomyositis was 44, 51, and 13, respectively. In pyogenic arthritis, the hip joint was the most commonly affected site (24 of 51 patients, 47.1%), where the femur was the most frequently involved site in osteomyelitis (14 of 44 patients, 31.8%). Among 13 patients with pyomyositis, 12 (92.3%) had muscle involvement around the hip joint, with the obturator muscles being the most commonly affected. Bacterial organisms were identified by venous blood or pus culture in 11 pyomyositis patients, with methicillin-sensitive *Staphylococcus aureus* (MSSA) detected in five cases. During the first 16 years of the study period, 43 patients were diagnosed with pyogenic arthritis or osteomyelitis, while only two cases of pyomyositis were recorded. In contrast, over the most recent 16 years, pyogenic arthritis and osteomyelitis cases increased to 53, while pyomyositis cases rose to 10.

Conclusions

At our institution, pediatric pyomyositis primarily affected the periarticular muscles of the hip. Although a rising trend in cases was observed, the difference between the earlier and later periods was not statistically significant (p=0.069). These findings emphasize the importance of including pyomyositis in the differential diagnosis of pyogenic hip infections and recommend early MRI evaluation prior to joint aspiration.

## Introduction

Pyogenic arthritis, osteomyelitis, and pyomyositis are bacterial infections characterized by inflammatory reactions and abscess formation. Pyogenic osteomyelitis is more common in children than in adults as it is influenced by the presence of the physis and the hemodynamics of the metaphysis. Its incidence ranges from 1.2 to 13 cases per 100,000 children [1]. The infection frequently extends to adjacent soft tissues, resulting in secondary pyogenic arthritis, which is a condition requiring prompt diagnosis and treatment in pediatric patients. Pyomyositis is a bacterial infection of skeletal muscle caused by hematogenous spread. Traditionally, it has been regarded as more common in tropical and subtropical climates [2-5]. However, since the 2000s, reports of its occurrence in temperate regions have increased [6], with numerous case reports of pyomyositis in children [7-14]. As a result, pyomyositis has become an important differential diagnosis among skeletal infectious diseases in children living in temperate areas. While some studies have described a rising trend of pyomyositis in several temperate countries [15-19], no studies have specifically examined this trend in Japan, and only case reports of pediatric pyomyositis have been published in Japanese. Therefore, we hypothesize that the incidence of pediatric pyomyositis is increasing in Japan, a country located in a temperate region. To investigate the current status of pediatric pyomyositis in this country, we analyzed cases of pyogenic arthritis, osteomyelitis, and pyomyositis over a 32-year period at our institution, which is located at approximately 35 degrees north latitude in a temperate region. Based on these findings, we report the characteristics of pediatric pyomyositis in Japan.

## Materials and methods

This study was approved by the Ethics Committee of Osaka Women's and Children's Hospital (approval number: 1799). Information about the study was made available on the hospital’s website.

Study subjects

We retrospectively analyzed 108 patients under the age of 15 who were diagnosed with pyogenic arthritis, pyogenic osteomyelitis, or pyomyositis and received follow-up care at the Department of Orthopedic Surgery, Osaka Women’s and Children’s Hospital, between April 1991 and March 2022. All patients underwent radiographic evaluation and magnetic resonance imaging (MRI). Clinical data, including radiographic and MRI findings, were collected from the hospital’s medical records by the first author.

Diagnosis criteria

The diagnosis was based on clinical symptoms such as local pain, fever, and pseudoparalysis, along with elevated inflammatory markers, neutrophilic leukocytosis, radiographic bone changes, magnetic resonance imaging (MRI) findings in bone, joint, or muscular tissues, and histological findings. MRI findings include joint effusion, bone destruction with abscess formation, and intramuscular abscesses. Pyogenic arthritis was diagnosed when joint effusion was present without accompanying bone or muscle abscess formation by MRI investigation. Osteomyelitis extending into adjacent joints was not classified as pyogenic arthritis. Similarly, pyomyositis secondary to osteomyelitis penetrating adjacent muscle tissue was classified as osteomyelitis, not as pyomyositis. A definitive diagnosis was established when the causative organism was identified through venous blood, pus, or tissue cultures. If no organism was identified, the diagnosis was determined through discussion based on clinical and imaging findings by at least two orthopedic surgeons. Patients with penetrating trauma or immunocompromising conditions were excluded from the study.

Analysis items

We reviewed patients' sex, age at onset, affected site, causative organism, and year of diagnosis. The onset date was defined as the day of the patient’s first visit to either our hospital or a referring institution. The characteristics of pyomyositis were then compared with those of pyogenic arthritis and osteomyelitis. In addition, the incidence of pyomyositis was assessed between the first 16 years and the most recent 16 years of this study period. 

Statistical analysis

Statistical analysis was performed using EZR (version 1.68). Differences in mean age at onset were assessed using one-way analysis of variance (ANOVA) test and Tukey's multiple comparison test, with p-values < 0.05 considered statistically significant. Differences in the incidence rates of skeletal infections and pyomyositis between the first 16 years and the most recent 16 years were analyzed using Fisher’s exact test, with a significance threshold of p-values < 0.05. Post hoc power analysis was also conducted using EZR (version 1.68).

## Results

A total of 108 patients with pyogenic arthritis, osteomyelitis, and pyomyositis were treated at our hospital until their infection was healed. All patients were followed until skeletal maturity to monitor for the development of skeletal deformities. This cohort included 51 patients with pyogenic arthritis, 44 with pyogenic osteomyelitis, and 13 with pyomyositis. The number of lost follow-up patients was 10 (19.6%) in pyogenic arthritis, 12 (27.3%) in osteomyelitis, and two (15.4%) in pyomyositis. Among patients with pyogenic arthritis, there were 26 male patients and 25 female patients, with a mean age at onset of 2.8 years (95% confidence interval (CI), 1.9-3.8). In pyogenic osteomyelitis, 29 male patients and 15 female patients were affected, with a mean age at onset of 4.5 years (95% CI, 3.1-5.8). Among those with pyomyositis, five male patients and eight female patients were diagnosed, with a mean age at onset of 5.4 years (95% CI, 2.7-8.1) (Table 1). There is no significant difference in the mean age at onset among three groups (p=0.11 between arthritis and osteomyelitis, p=0.10 between arthritis and pyomyositis, and p=0.76 between osteomyelitis and pyomyositis).

**Table 1:** Mean age at onset and sex in pyogenic arthritis, pyogenic osteomyelitis, and pyomyositis

	Arthritis	Osteomyelitis	Pyomyositis
Mean Age at Onset (years)	2.8±3.4(0.0-11.4)	4.5±4.4 (0.0-14.8)	5.4±4.5 (0.0-12.0)
Sex (Male:Female)	26:25	29:15	5:8

**Figure 1:**
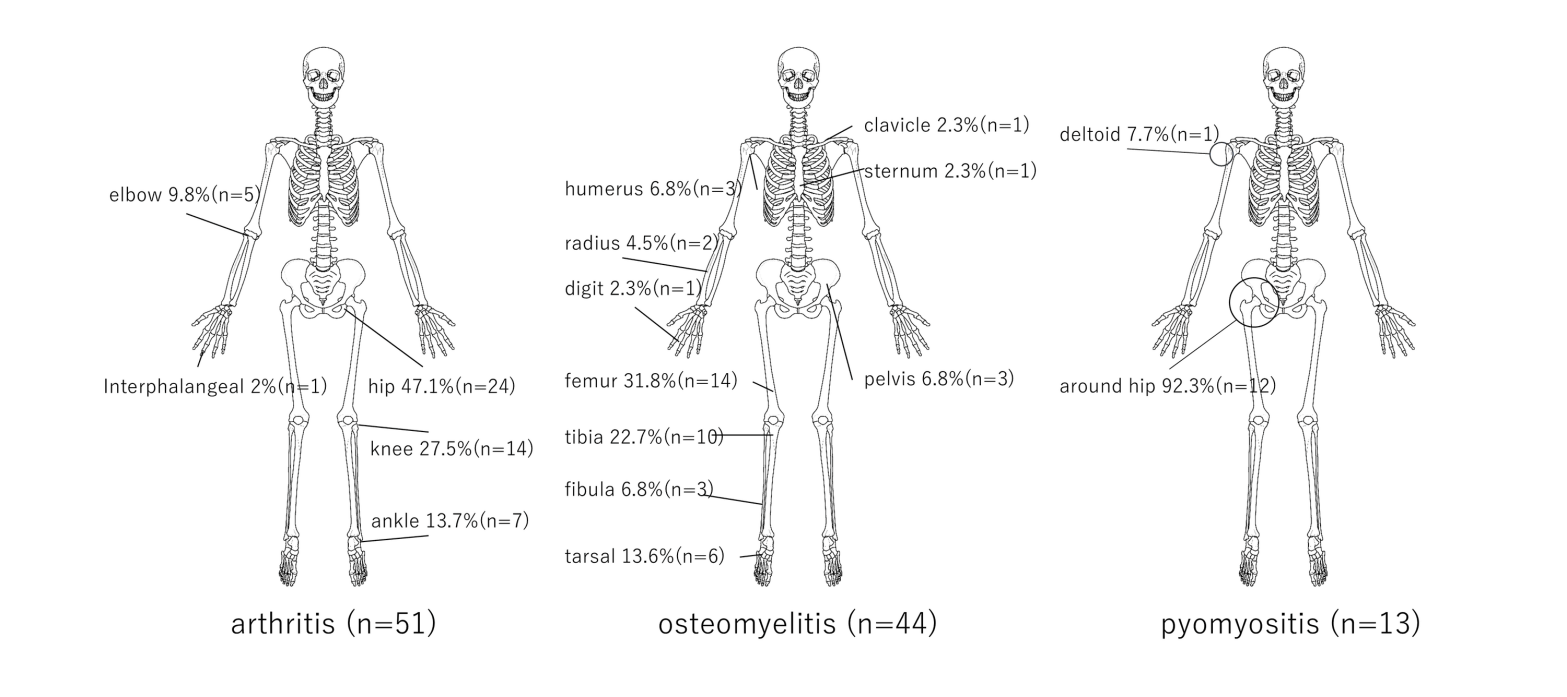
Affected site of pyogenic arthritis, pyogenic osteomyelitis, and pyomyositis

In pyogenic arthritis, the hip joint was the most commonly affected site (24 cases, 47.1%), followed by the knee (14 cases, 27.5%) and ankle (seven cases, 13.7%). In pyogenic osteomyelitis, the femur was the most frequently involved bone (14 cases, 31.8%), followed by the tibia (10 cases, 22.7%). Among the 13 patients with pyomyositis, all but one (92.3%) had muscle involvement around the hip joint, with the obturator muscles being the most commonly affected (Figure 1).

Table 2 summarizes the mean age at onset and the age distribution by affected site for each disease. The mean age at onset for patients with pyomyositis around the hip was 5.8 years (range: 0.0-12.0), which was older than that for patients with hip arthritis (2.7 years) or femoral osteomyelitis (2.7 years). In contrast, all patients with pelvic osteomyelitis had disease onset during their teenage years. Regarding the mean age at onset for cases involving the hip region (hip in arthritis, femur in osteomyelitis, around hip in pyomyositis), there was a significant difference between the arthritis and pyomyositis groups (p=0.03), while no significant differences were observed between the arthritis and osteomyelitis groups (p=0.99) or the osteomyelitis and pyomyositis (p=0.06). 

**Table 2:** Mean age and age distribution at onset per site of pyogenic arthritis, pyogenic osteomyelitis, and pyomyositis

Arthritis		Osteomyelitis		Pyomyositis	
	Mean age (distribution) (years)		Mean age (distribution) (years)		Mean age (distribution) (years)
Hip	2.7(0.0-11.3)	Femur	2.7(0.0-7.3)	Around hip	5.8(0.0-12.0)
Knee	2.5(0.0-9.5)	Tibia	7.6(0.1-11.7)	Deltoid	0.0(0.0)
Ankle	5.0(0.0-11.3)	Foot	3.3(0.0-7.5)		
Elbow	2.8(0.1-7.5)	Humerus	0.4(0.1-1.0)		
Finger	0.3(0.3)	Fibula	5.3(1.0-8.2)		
		Pelvis	12.1(11.4-13.1)		
		Others	1.9(0.1-5.7)		

The exception was a patient with upper arm involvement, who was suspected to have acquired a vertical *Streptococcus agalactiae* infection at birth. Causative organisms were not identified through culture tests (blood or pus culture) in 27 patients with pyogenic arthritis (52.9%) and 18 with osteomyelitis (40.9%). Among identified pathogens, methicillin-sensitive *Staphylococcus aureus* (MSSA) was detected in 11 patients with pyogenic arthritis (21.6%) and 10 patients with osteomyelitis (22.7%), methicillin-resistant *Staphylococcus aureus* (MRSA) was detected in five patients with arthritis (9.8%) and nine patients with osteomyelitis (20.5%), *Mycobacterium tuberculosis *was detected in three patients with pyogenic arthritis (5.9%) and four with osteomyelitis (9.1%), *Streptococcus* was detected in three patients with arthritis or osteomyelitis, *Haemophilus parainfluenzae* was detected in two patients with pyogenic arthritis (3.9%), and methicillin-resistant *Staphylococcus epidermidis *(MRSE) in one osteomyelitis patient (2.8%), *Pseudomonas aeruginosa* in and *Salmonella* in one pyogenic arthritis patient (2.0%) each. In pyomyositis, bacterial organisms were identified through venous blood or abscess culture in 11 patients (84.6%). These included MSSA in five patients (38.5%), MRSA in three patients (23.1%), *Streptococcus agalactiae*, *Streptococcus pneumoniae*, and *Salmonella* in one patient (7.7%) each. Two patients with no detection of causative organisms had received antibiotic therapy at previous hospitals before the initiation of our treatment (Table 3).

**Table 3:** Causative organisms of pyogenic arthritis, osteomyelitis, and pyomyositis

Causative organisms	Arthritis	Osteomyelitis	Pyomyositis
Methicillin-sensitive *Staphylococcus aureus *(MSSA)	21.6% (n=11)	22.7% (n=10)	38.5% (n=5)
Methicillin-resistant *Staphylococcus aureus *(MRSA)	9.8% (n=5)	20.5% (n=9)	23.1% (n=3)
Methicillin-resistant *Staphylococcus epidermidis *(MRSE)		2.8% (n=1)	
Streptococcus pyogenes	2.0% (n=1)	2.8% (n=1)	
Streptococcus agalactiae		2.8% (n=1)	7.7% (n=1)
Streptococcus pneumoniae			7.7% (n=1)
Haemophilus parainfluenzae	3.9% (n=2)		
Pseudomonas aerginosa	2.0% (n=1)		
Mycobacterium tuberculosis	5.9% (n=3)	9.1% (n=4)	
Salmonella	2.0% (n=1)		7.7% (n=1)
Not detected	52.9% (n=27)	40.9% (n=18)	15.4% (n=2)
Total	100% (n=51)	100% (n=44)	100% (n=13)

**Figure 2:**
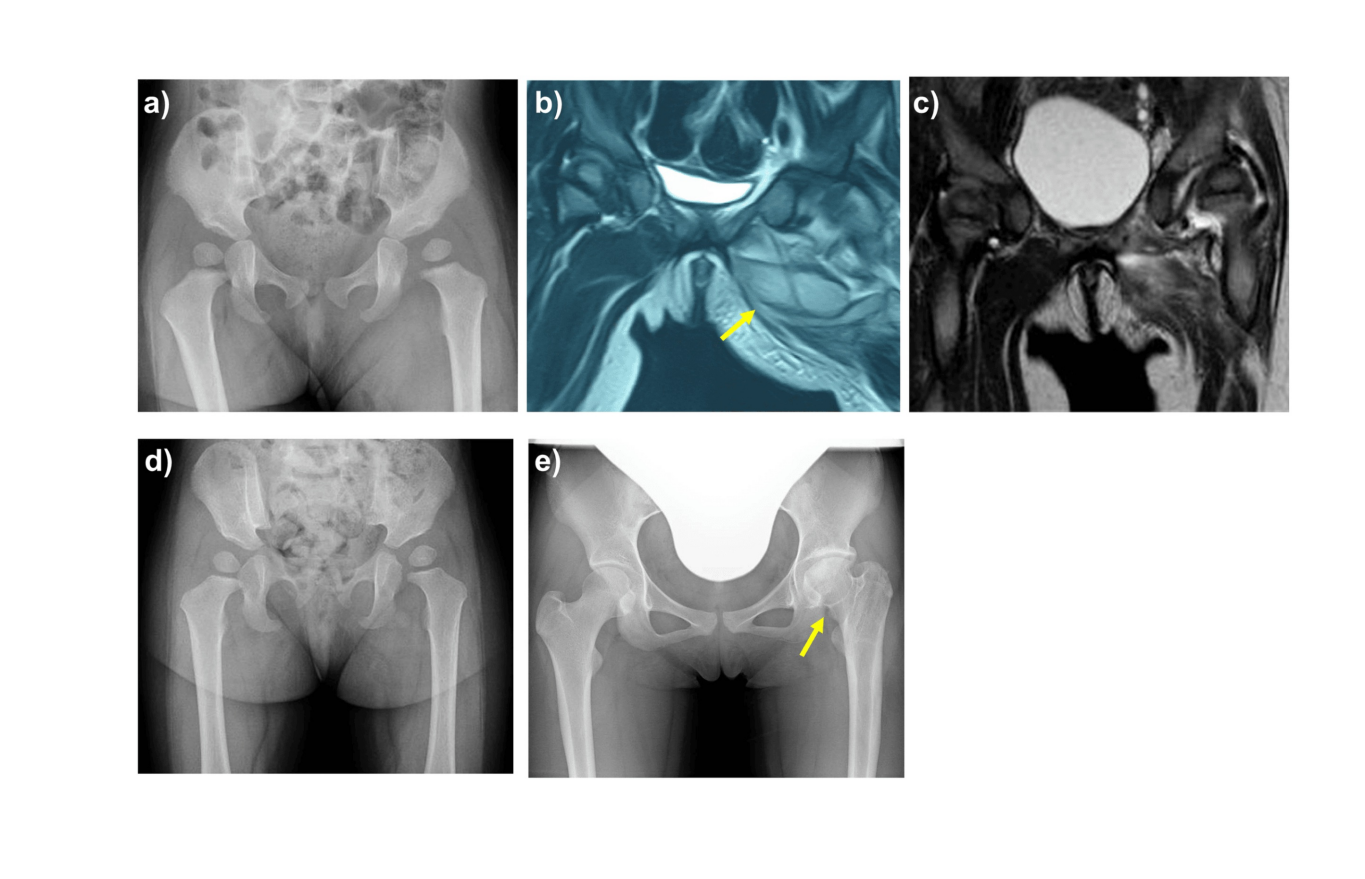
A case of secondary pyogenic arthritis due to pus infiltration into the joint following aspiration a) Lateralization of the left femoral head before treatment.  b) Abscess formation in adductors of the left hip (yellow arrow) in MRI.  c) Joint fluid in the left hip joint after operation of abscess drainage. d) Normalization of the left hip after operation of joint irrigation. e) Femoral head and short neck deformities of the left hip joint (yellow arrow) at the final examination.

Among all patients with pyomyositis around the hip joint, pyogenic arthritis was initially suspected based on physical examination. Arthrocentesis was performed in two patients with pyomyositis, but joint fluid cultures were negative. One of these patients subsequently developed secondary pyogenic arthritis due to pus infiltration into the joint following aspiration, leading to hip joint deformity (Figure 2).

**Figure 3:**
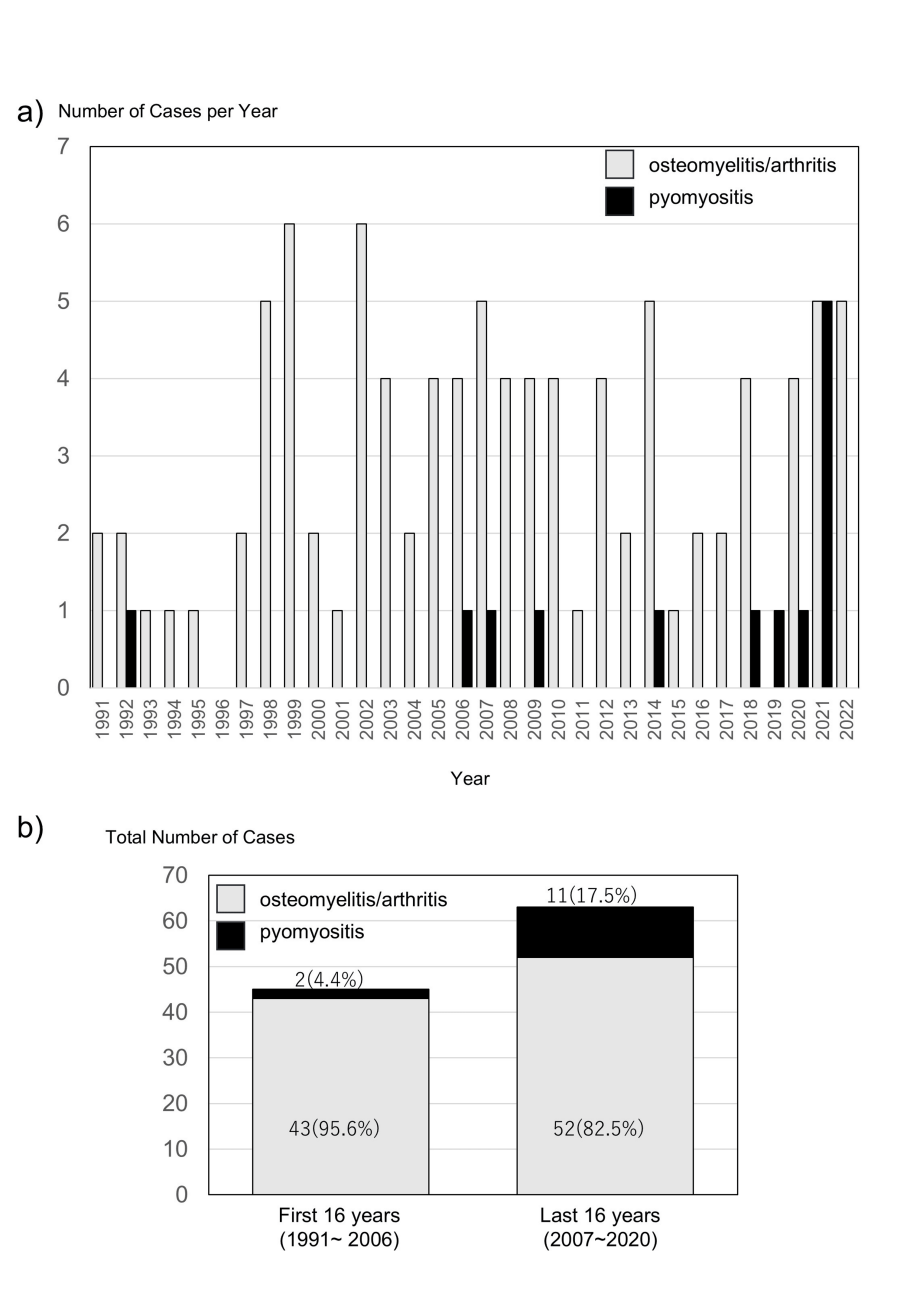
Number of cases with osteomyelitis/arthritis or pyomyositis a) Number of cases with osteomyelitis/arthritis or pyomyositis per year. b) Total number of cases with osteomyelitis/arthritis or pyomyositis for the first 16 years (1991~2006) and the last 16 years (2007~2022).

All skeletal infections accounted for 0.07% of all pediatric admissions of our hospital in the first 16 years and 0.05% in the most recent 16 years. There is no significant difference in the skeletal infection rate of two periods (p-value of 0.229, power of 100%). Figure 3a shows the annual number of patients diagnosed with pyogenic arthritis/osteomyelitis and pyomyositis. During the first 16 years, 43 cases of pyogenic arthritis/osteomyelitis were recorded, while only two cases of pyomyositis were identified. One of these cases was suspected to have resulted from maternal-to-neonatal transmission. Pyomyositis accounted for 4.4% of all bone, joint, and muscle infections during this period. In contrast, during the most recent 16 years, 52 cases of pyogenic arthritis/osteomyelitis and 11 cases of pyomyositis were recorded, with pyomyositis comprising 17.5% of such infections (Figure 3b). The difference in the incidence of pyomyositis in skeletal infection between the two groups was evaluated using Fisher’s exact test with a resulting p-value of 0.069 and using post hoc power analysis with a resulting power-value of 40％.

## Discussion

Infectious diseases of the skeletal system can lead to the destruction of bones, joints, and soft tissues such as muscles and tendons. These conditions can impair activities of daily living, and such functional disturbances may be prolonged, especially in children. The incidence of osteomyelitis ranges from 1.2 to 13 cases per 100,000 children per year [1], while that of pyogenic arthritis has been reported as 3.28 cases per 100,000 children in the United States [20]. According to an analysis of 286 children with osteoarticular infections by *Staphylococcus aureus*, 9% developed musculoskeletal complications [21]. To prevent such complications, empirical antibiotic therapy is often initiated prior to a definitive diagnosis.

Pyomyositis is another infectious disease affecting skeletal components. However, it remains relatively poorly understood among orthopedic surgeons. This disease, which is sometimes referred to as tropical pyomyositis, is more common in tropical regions of Asia, Africa, Oceania, and the Caribbean islands, where it accounts for approximately 1% to 4% of all hospital admissions [22]. In tropical regions, it predominantly affects otherwise healthy young children (aged 2 to 5 years) and adults (aged 20 to 45 years). In contrast, in temperate countries, pyomyositis is more frequently observed in immunocompromised or critically ill adults [6,23,24], and most cases in these regions tend to occur during warmer months or rainy seasons. Although previously considered rare in temperate climates, several case reports have documented the occurrence of pyomyositis in children, and multiple studies have reported an increase in pediatric cases in the United Kingdom, the United States, and Australia [14-16,18,19], which are considered temperate regions, since 2015. The incidence of pediatric pyomyositis in temperate regions has been estimated at up to 0.1% of pediatric hospital admissions [6,15,16], with one or two cases occurring per 4,000 pediatric admissions in the United States [25]. In this study, we analyzed the characteristics and incidence trends of pediatric pyomyositis during 32 years at a single institution in Japan. Our findings revealed an increasing number of pyomyositis cases among children, a tendency for the condition to occur more frequently around the hip joint, and that the causative organisms were largely similar to those responsible for pyogenic osteomyelitis and septic arthritis. 

In our institution, pediatric pyomyositis cases were not observed in the 1990s, except for a single case with confirmed vertical transmission, and no cases were recorded during the first half of the 2000s. However, cases began to emerge in the late 2000s, and in recent years, they have been recorded annually. Kiran et al. reported an increase in peri-pelvic pyomyositis cases in the United Kingdom since 2014 [18], while Moriarty et al. documented a gradual rise in cases in Queensland, Australia, since the early 2000s [16]. These studies indicate an increasing incidence of pyomyositis in temperate regions, consistent with our findings at a single institution in a similar climate. Over the most recent 16 years, the number of pediatric pyomyositis cases was five times higher than that in the first 16 years. In contrast, the number of pyogenic arthritis and osteomyelitis cases increased by only 1.23-fold over the same timeframe. These findings suggest a possible rise in the incidence of pediatric pyomyositis. However, the difference in incidence between the two periods was not statistically significant, as evaluated by Fisher’s exact test (p = 0.069). Given this p-value, a significant increase may become evident with a longer observation period or a larger sample size. 

Regarding the focus of infection in pyomyositis, the peri-hip muscles were affected in 12 cases (92.3%), whereas osteomyelitis and arthritis more commonly involved the areas around the hip joint. Our findings are consistent with those of Kiran et al., who reported that pyomyositis around the hip required differentiation from pyogenic hip arthritis in 35 of 41 cases (85%) [18]. Additionally, numerous studies have documented cases of pelvic pyomyositis or periarticular pyomyositis of the hip [26-28]. In our study, initial diagnoses by previous physicians included pyogenic hip arthritis, leading to joint aspiration in two cases. In one of these cases, iatrogenic pyogenic arthritis likely developed due to the introduction of pus into the joint during arthrocentesis, ultimately resulting in secondary hip deformity. Therefore, when orthopedic surgeons suspect an inflammatory condition around the hip joint, such as pyogenic arthritis or transient synovitis, MRI should be the first-line imaging modality before performing joint aspiration for definitive diagnosis. Several studies have highlighted the utility of MRI in diagnosing pyomyositis [18,29,30]. We strongly recommend MRI to prevent bacterial contamination of the joint caused by arthrocentesis, thereby reducing the risk of iatrogenic pyogenic arthritis. Furthermore, contrast-enhanced MRI is especially valuable in identifying the extent of the lesion, the site of abscess formation, and the presence of severe synovitis. However, the cost-effectiveness of MRI in the diagnosis and management of pyogenic musculoskeletal diseases remains a significant concern. In Japan, pediatric patients fortunately incur little or no out-of-pocket medical expenses due to the universal health insurance system and additional support from local governments. Therefore, pediatric orthopedic surgeons can readily utilize MRI in clinical practice. In settings where MRI is not always accessible, it may be reasonable to reserve its use for cases with suspected skeletal infections around the hip joint, given the high prevalence of pyomyositis in this area and the need for differential diagnosis. In such cases, ultrasonography can serve as a useful alternative. However, ultrasonographic evaluation may occasionally fail to visualize the entire lesion, especially when multiple muscle groups are involved or when the affected area is large. From the perspective of comprehensive anatomical assessment, MRI is superior to ultrasonography. In addition, our study identified a significant difference in the mean age at onset between patients with pyogenic hip arthritis and those with pyomyositis around the hip (2.8 years vs. 5.4 years, respectively). This age difference may serve as a useful indicator in the differential diagnosis of hip-related infections in children. 

The most common bacterial pathogen in all three pyogenic diseases was *Staphylococcus aureus*. The prevalence of *Staphylococcus aureus*-induced infections was 36.8% (35 of 95 cases) in pyogenic arthritis/osteomyelitis and 61.6% (8 of 13 cases) in pyomyositis. Similarly, Moriarty et al. reported that *Staphylococcus aureus *was identified in nearly half of their pyomyositis cases [16], and other studies have also demonstrated its predominance in pyomyositis [15,18]. Although our study is limited by a small sample size, the observed frequency is consistent with these previous reports. Therefore, while identifying the causative organism remains essential, empirical treatment for pyomyositis should initially target *Staphylococcus aureus*. 

This study has a major limitation. The sample size of our study is small and based on data collected from a single institution in Japan. Our hospital is the largest pediatric center in Osaka and serves as a key facility for surgical intervention in the southern medical region of Osaka, which had approximately 365,000 children under the age of 15 in 2022. Therefore, nearly all pediatric patients with skeletal infections in this area were likely referred to our hospital, allowing for a reasonable estimation of the incidence of pyogenic skeletal diseases within this region. However, this study could not capture cases of mild pyomyositis that were managed conservatively with oral antibiotics in general pediatric outpatient clinics and thus were not referred to our hospital. Furthermore, while the population of children under 15 in Osaka was about one million, the nationwide pediatric population in Japan was approximately 14.65 million in the same year. Therefore, we are unable to draw definitive conclusions regarding national trends in the incidence of pyomyositis among Japanese children. To better understand the epidemiological and clinical characteristics of pediatric pyomyositis in Japan, a larger, multicenter study is warranted. One potential approach would be to propose a nationwide surveillance system for pyogenic skeletal diseases through the Japanese Pediatric Orthopaedic Association, which includes the majority of pediatric orthopedic surgeons working at pediatric hospitals across the country.

## Conclusions

Our findings suggest the possibility of a rising trend in pediatric pyomyositis cases in Japan, similar to trends observed in other temperate countries. This result should be corroborative by further research such as Japanese multicenter studies because the sample size of our study is small and based on data from a single institution. However, given that the affected sites and causative organisms of pyomyositis do not differ significantly from those of pyogenic arthritis and osteomyelitis, pediatric orthopedic surgeons in the temperate climates including Japan should consider pyomyositis as a differential diagnosis when evaluating pediatric pyogenic diseases of the musculoskeletal system, particularly in patients around the hip joint without joint effusion. When the situation allows, MRI is a useful modality for the differential diagnosis. 
